# Commentary on: Changes in Glucose Control and Lipid Levels Following Trunk-Based Body Contouring Surgery in Postbariatric and Nonbariatric Patients

**DOI:** 10.1093/asjof/ojad001

**Published:** 2023-01-07

**Authors:** M Shuja Shafqat, Kevin H Small

**Affiliations:** private practice in Roslyn Heights, NY, USA; private practice in Roslyn Heights, NY, USA

See the Original Article here.

The effect of weight loss and bariatric surgery on comorbidities is well documented. In this article, the authors look at the effects of trunk-based body contouring (BC) surgery on metabolic changes, specifically on glucose and lipid levels, in nonbariatric and bariatric surgery patients.^1^ The results of the study can assist with preoperative patient counseling and highlight the need for further study in this area (Video).

The authors note a limitation that they do not have a bariatric only comparable cohort. They also note that their postbariatric cohort of BC patients regained significantly more weight than nonbariatric patients which would affect the results of this study. This contrasts with other studies which show postbariatric BC patients have improved % total weight loss.^2,3^ In addition, most of the patients still had higher BMI, gastric bypass over sleeve gastrectomy, and none had posterior contouring which can remove large excess fat stores in the flanks,^4^ all of which would further affect weight control and metabolism.

We believe this is an important study to demonstrate that BC procedures removing skin and subcutaneous fat may not have the same metabolic effect as surgical or nonsurgical weight loss^5^; however, further study is needed. This is important when counseling patients about these procedures.

## Supplementary Material

ojad001_Supplementary_Data
